# Good News for Cabbageheads: Controlling *Phelipanche aegyptiaca* Infestation under Hydroponic and Field Conditions

**DOI:** 10.3390/plants11091107

**Published:** 2022-04-19

**Authors:** Amit Wallach, Guy Achdari, Hanan Eizenberg

**Affiliations:** 1 Department of Phytopathology and Weed Research, Agricultural Research Organization, Newe Ya’ar Research Center, Ramat Yishay 3009503, Israel; achdarig@volcani.agri.gov.il (G.A.); eizenber@volcani.agri.gov.il (H.E.); 2 The Robert H. Smith Institute of Plant Sciences and Genetics, The Robert H. Smith Faculty of Agriculture, Food and Environment, The Hebrew University of Jerusalem, Rehovot 7610001, Israel

**Keywords:** *Phelipanche aegyptiaca*, glyphosate, ethametsulfuron-methyl, chemical control

## Abstract

*Phelipanche aegyptiaca* (Orobanchaceae) is a parasitic weed that causes severe yield losses in field crops around the world. After establishing vascular connections to the host plant roots, *P. aegyptiaca* becomes a major sink that draws nutrients, minerals, and water from the host, resulting in extensive crop damage. One of the most effective ways to manage *P. aegyptiaca* infestations is through the use of herbicides. Our main objective was to optimize the dose and application protocol of herbicides that effectively control *P. aegyptiaca* but do not damage the cabbage crop. The interactions between the cabbage roots and the parasite were first examined in a hydroponic system to investigate the effect of herbicides on initial parasitism stages, e.g., germination, attachment, and tubercles production. Thereafter, the efficacy of glyphosate and ethametsulfuron-methyl in controlling *P. aegyptiaca* was examined in five cabbage fields naturally infested with *P. aegyptiaca*. The herbicides glyphosate and ethametsulfuron-methyl were applied on cabbage foliage and in the soil solution, both before and after the parasite had attached to the host roots. A hormesis effect was observed when glyphosate was applied at a dose of 36 g ae ha^−1^ in a non-infested *P. aegyptiaca* field. Three sequential herbicide applications (21, 35, and 49 days after planting) effectively controlled *P. aegyptiaca* without damaging the cabbages at a dose of 72 g ae ha^−1^ for glyphosate and at all the examined doses for ethametsulfuron-methyl. Parasite control with ethametsulfuron-methyl was also effective when overhead irrigation was applied after the herbicide application.

## 1. Introduction

Egyptian broomrape *Phelipanche aegyptiaca* (Orobanchaceae) is a root parasitic plant that has a wide range of hosts in field crops. As such, it is considered a major troublesome weed that causes severe yield losses around the world [1]. *P. aegyptiaca* is very common in the Mediterranean region and East Asia, and there are also reports that it has taken hold in parts of Australia, Europe, and the Americas [2]. Ambient conditions in Israel, with its Mediterranean climate, support the development of *P. aegyptiaca* in vegetable fields [3]. For all the broomrapes (*Phelipanche* and *Orobanche* spp.), including *P. aegyptiaca*, the seeds can lie dormant in the soil for a long time, even decades, and then germinate after pre-conditioning in response to germination stimulants [4,5]. After seed germination, the parasitic plant invades the host’s root vascular system, and once established, the parasite draws all its nutritional and water requirements from the host, resulting in extensive damage to the host plant.

One of the most effective ways to manage *P. aegyptiaca* infestations in the field is via the application of herbicides. However, the herbicide-based control of *P. aegyptiaca* in the field is a complex task, for two main reasons: the herbicide must be selective, i.e., not damage the host plants (since it will move through the host to the parasitic plant), and the types of herbicides that can be applied to the parasitic broomrapes are limited to particular target sites, e.g., photosystem inhibitors are not effective because of the absence of PSII in the parasitic plants [5]. Herbicides based on one of two modes of action are thus used to control *P. aegyptiaca*, i.e., through the inhibition of aromatic amino acid biosynthesis (by targeting 5-enolpyruvylshikimate-3-phosphate synthase (EPSPS); group 9 HRAC), e.g., glyphosate, which is generally applied to the foliage, or through the inhibition of branched-chain amino acid biosynthesis (by targeting acetolactate synthase (AHAS/ALS); group 2 HARC), e.g., imidazolinones, sulfonylureas, and pyrithiobac-sodium, which are usually applied to the soil for absorption by the roots. Therefore, the site of herbicide application must be chosen according to its mode of action [5].

In the proposed study, in which we investigated herbicides suitable for application in *P. aegyptiaca*-infested cabbage fields, we were in a position to leverage the knowledge acquired over the years by the Eizenberg group in the management of broomrape infestations of a variety of crop species [5], as reviewed in brief below. Goldwasser et al. (2001) studied the control of *P. aegyptiaca* parasitism in potato fields by the sequential application of the ALS inhibitors imazapic and rimsulfuron. They found that three sequential applications of imazapic on potato foliage 20, 40, and 60 days after full potato emergence effectively controlled *P*. *ramosa* but impaired the quality of the potatoes. By contrast, the application of rimsulfuron followed by overhead irrigation controlled *P. aegyptiaca* efficiently and did not harm the potatoes. The difference in response to the two herbicides may derive from the availability of rimsulfuron in the rhizosphere, which allows the direct translocation of the herbicide to the *P. aegyptiaca* attachments and may, therefore, prevent crop damage [6].

Cochavi et al. [7] developed a protocol to control *P. aegyptiaca* in carrot fields using foliar herbicide applications. They found that glyphosate and imazapic in doses lower than 108 g ae ha^−1^ and 2.4 g ai ha^−1^, respectively, did not harm the carrot taproot biomass in non-infested fields at low doses of herbicide. They also found that the yields of the carrots classified as class A were higher for sequential applications of glyphosate vs. imazapic. By contrast, imazamox at all examined doses led to a reduction in carrot yield. Therefore, they decided to continue the experiment with only glyphosate applied to the foliage by monitoring *P. aegyptiaca* development using a minirhizotron system when *P. aegyptiaca* attachments were 2 mm, and sequential applications were within 21 days intervals. When the carrot biomass was determined, a hormesis response was observed at a glyphosate concentration of 137 g ae ha^−1^ in a *P. aegyptiaca*-infested carrot field. However, three sequential applications of glyphosate at low doses of 54 or 108 g ae ha^−1^ were successful in controlling *P. aegyptiaca* [7].

Aly et al. (2001) conducted a study aiming to control *O. cumana* (sunflower broomrape) in the field. They found that sunflower vigor was impaired by two sequential applications of imazapic on sunflower foliage 12 cm and 55 cm tall with treatments at doses of 3.6 or 4.8 g ai ha^−1^. However, when they used drip irrigation and reduced the imazapic dose, the herbicide still gave effective control, facilitating an increase in the yield [8]. Eizenberg [9] also cooperated with a group in Oregon, USA [10], in a study aiming to control small broomrape (*O. minor*) in red clover fields. Using a growing degree days (GDD) model, they examined the effect of imazamox at 800 and 1000 GDD and found that all the examined doses controlled *O. minor* effectively, with the largest attachments being better controlled than the small attachments.

Some 20 years later, Eizenberg and Goldwasser [11] developed a holistic decision support system (DSS), which they designated ‘PICKIT’, to control *P. aegyptiaca* in fields of processing tomatoes [12]. The *P. aegyptiaca* management tools of the DSS are based on parasitism dynamics models [12,13] that estimate key parasitism stages in terms of GDD, foliar herbicide applications, and drip chemigation and then provide the optimal timing, doses, and methodology of herbicide application. Finally, Cohen et al. used GIS to approximate and evaluate the *P. aegyptiaca* infestation level in the field and on a regional scale [14].

Building on the knowledge acquired by the Eizenberg group, the objectives of the current study were twofold: (i) to investigate the effect of herbicides belonging to two different classes of compounds, glyphosate and ethametsulfuron-methyl (EMS), on cabbage yield; (ii) to optimize the dosage and application practice of the herbicides to effectively control *P. aegyptiaca* in cabbage fields. Both herbicides are considered appropriate for use in cabbage.

## 2. Materials and Methods

### 2.1. Plant Material

#### 2.1.1. Cabbage

The white cabbage (*Brassica oleracea*) cultivars ‘Froctor’ (Zeraim Gedera-Syngenta, Israel) ‘Fresco‘, and ‘Cheers’ (Eden Seeds, Moshav Hatzav, Israel) and the red cabbage cultivar ‘Grand-Rio’ (Tarsis Agrichem, Petah Tikvah, Israel) were used in this study. All cabbage plants that were used in the experiments were planted out 30 days after seeding in trays.

#### 2.1.2. Phelipanche Aegyptiaca

*P. aegyptiaca* inflorescences were collected in 2017 from a cabbage field in southern Israel. After the seeds had been sieved through a 300-micron mesh, they were stored at 4 °C in the dark until use.

### 2.2. Laboratory and Field Experiments

#### 2.2.1. Herbicides 

Glyphosate (Roundup^TM^, 360 g ae /L) was obtained from Bayer, Monsanto Company (St. Louis, MO, USA), and ethametsulfuron-methyl (Salsa^TM^ 75% WG) from Du Pont Inc. (Wilmington, DE, USA). EMS was mixed with alkylaryl polyether alcohol (DX, 800 g/L; Adama-Agan, Ashdod, Israel).

#### 2.2.2. Germination Test

*P. aegyptiaca* seeds were surface-sterilized for 3 min in 70% ethanol and then for 10 min in 1% sodium hypochlorite before washing with distilled water [15]. The seeds were kept in a sterile hood chamber until dry and then stored at 4 °C in the dark until use. For the germination experiment, the seeds were spread on 8 mm Whatman^®^ glass microfiber filter disks, Grade GF/A (Whatman International, Maidstone, UK), held in Petri dishes. The disks were wetted with 31 µL of distilled water, and the Petri dishes were sealed with Parafilm strips. The experiment was conducted in a chamber held in the dark at 25 °C. After 7 days of preconditioning, 28 µL of GR_24_ (StrigoLab, Turin, Italy) at a concentration of 10^−6^ µL/mL was added to each disk; demineralized water was used as the control. Four days after GR_24_ was added, *P. aegyptiaca* germination was determined with a binocular electronic microscope (Leica M80, Wetzlar, Germany) [16].

#### 2.2.3. Experiments in Polyethylene Bags

In addition to the above experiments, experiments in polyethylene bags (PEB) were performed to investigate *P. aegyptiaca* herbicide response at the subsurface stages, both pre- and post-attachment to the host’s roots, according to the method described by Parker and Dixon [17]. Briefly, cabbage (cultivar ‘Froctor’) seedlings were re-rooted in 250 mL of 5% Hoagland’s solution [18]. When the seedlings had developed new root systems, the plants were placed on 35 × 24 mm GF/A glass microfiber paper sheets, and *P. aegyptiaca* seeds were spread uniformly over the paper sheets [17]. Thereafter, 1 mL of herbicide at a concentration of 5 × 10^−3^ mL/L for glyphosate or 0.125 g/L for EMS was applied using a manual sprinkler over the cabbage foliage immediately after the parasite was attached to the host. Three and six days after herbicide application, healthy attachments were counted and classified according to Perez-de-Luque et al. (2016) [19]. Control efficacy is presented as a percentage of total healthy attachments of the non-treated control.

When herbicide was applied in the pre-attachment parasitism stages, 1.5 mL of glyphosate or EMS solution was applied as described above or by syringing 5 mL of EMS onto the GF/A paper sheet. When the herbicide was applied in the pre-attachment parasitism experiment, *P. aegyptiaca* seeds were counted seven days after herbicide application, and *P. aegyptiaca* establishment was assessed as a percent of the necrotic attachments out of the total number of attachments.

#### 2.2.4. Field Experiments

Seven field experiments were conducted in commercial cabbage fields, some naturally infested with *P. aegyptiaca* and others not infested, at different locations in Israel—Sde Tzvi (experiments designated A, B, and D), Nahalal (experiment C), Beit Hagedi (experiments E and F), and Mevo Hama (experiment G). The experiments were conducted in blocks in a random factorial design. Each plot was 5 m long and 1.93 m wide. The herbicides were applied sequentially 21, 35, and 49 days after planting (DAP) at 200 L ha^−1^ with a motorized GKS15 sprayer equipped with a Tee Jet^®^ 110015 nozzle (Maruyama Mfg CO. Inc., Chiyoda-ku, Japan) and operated at a pressure of 300 kPa. In experiments A and B, each herbicide was tested at six different doses for foliar application as follows: glyphosate 36, 72, 144, 188, 432, and 576 g ae ha^−1^ and EMS 4, 9, 18, 28, 37, and 75 g ai ha^−1^. The control plots were not treated with herbicide. In experiments C, D, E, and F, glyphosate and EMS were tested for a foliar application on the basis of the doses that were found in experiments A and B, namely, 72 g ae ha^−1^ and 18.5 g ai ha^−1^, respectively. In experiment G, 300 m^3^ ha^−1^ water was applied with overhead irrigation after each herbicide application at 21, 35, and 49 DAP within eight hours after herbicide application. At the conclusion of the field experiments, cabbage heads were harvested from an area of 4 m^2^ in each plot.

### 2.3. Statistical Analyses

All data sets were analyzed using RStudio Version 1.4.1717 (RStudio team). ANOVA was performed if the data showed a normal distribution. The normality of the data was determined using the Shapiro–Wilk test, and the means were compared using Tukey’s honestly significant difference (HSD) (*p* < 0.05). Data sets with non-normal distributions were analyzed using a general linear model (GLM), and least-squares means were computed into the package “EMMEANS” [20]. Means in the PEB experiment three and six days after herbicide application were compared using a paired *t*-test.

Dose–response (hormesis) was incorporated into the “drc” [21] in R as follows:

(1) $$y=c+\frac{\left(d-c+fx\right)}{1+exp\left(b\left(log\left(x\right)-log\left(e\right)\right)\right)}$$

*b* and *e* have no direct interpretation (while *b* and *e* are constants), *c* represents the lower horizontal asymptote, *d* represents the upper horizontal asymptote, and *f* is the size of the hormesis effect. The resulting model is a four-parameter log-logistic model [22].

## 3. Results

### 3.1. Effect of Herbicides on P. aegyptiaca in Polyethylene Bags

#### 3.1.1. Herbicide Application Post-Attachment

**Figure 1:**
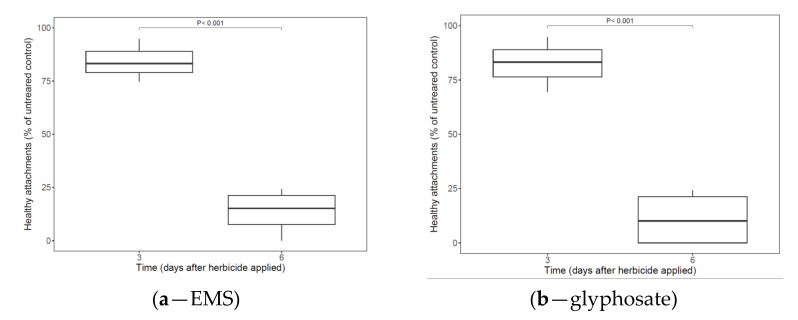
Healthy attachments of *P. aegyptiaca* compared with non-treated control in cabbage grown hydroponically in polyethylene bags. Ethametsulfuron-methyl (EMS, **a**) and glyphosate (**b**) were applied to the cabbage foliage after *P. aegyptiaca* attachment. Means were compared using a paired *t*-test (*p < 0.05*); the numbers in the figure represent *p*-values.

Effective control (vs. untreated cabbage plants) was obtained by foliar spraying of *P. aegyptiaca*-infected cabbages (growing under hydroponic conditions) with glyphosate or EMS when the broomrape tubercles reached 2.5 mm in size (Figure 1). For the herbicide-treated plants, there were 89% and 91% healthy *P. aegyptiaca* attachments for glyphosate and EMS, respectively, three days after the treatment, compared with 27% and 29% healthy attachments, respectively, six days after the treatment (Figure 1).

#### 3.1.2. Herbicide Applications Pre-Attachment

**Figure 2:**
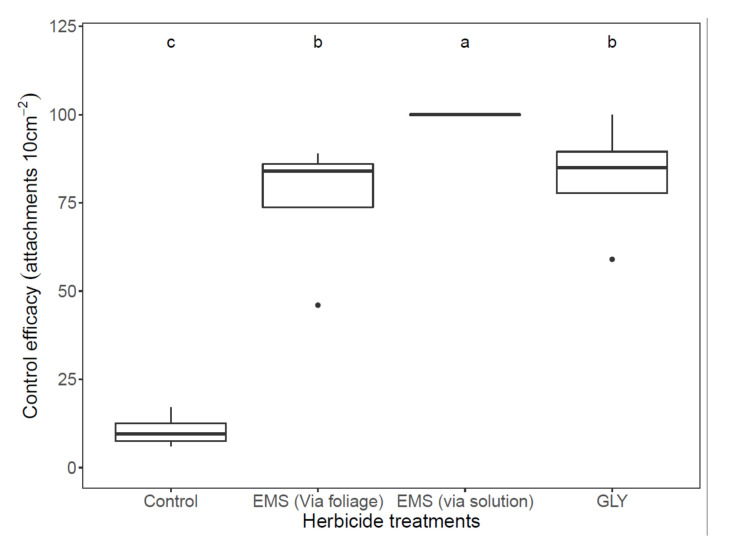
Control efficacy of *P. aegyptiaca* in cabbage grown hydroponically in polyethylene bags by applying the herbicide, glyphosate (GLY), or ethametsulfuron-methyl (EMS), before *P. aegyptiaca* attachment to the cabbage roots (vs. the non-treated control). Means were compared using GLM. (*p* < 0.05); means labeled with the same letter do not show significant differences.

When herbicides were applied in the pre-parasitism stage, both glyphosate and EMS markedly impaired *P. aegyptiaca* attachment (Figure 2). Excellent control of *P. aegyptiaca* was achieved by the injection of EMS into the root zone, namely, 100% of the seeds showed necrosis. For the foliar application of glyphosate and EMS, 82% and 75%, respectively, of seeds showed necrosis, compared with 10% necrotic seeds in the non-treated control (Figure 2).

### 3.2. Field Experiments

The seven experiments that were conducted under field conditions aimed to determine the optimal herbicide doses that would not damage the cabbages (in the fields not infested with *P. aegyptiaca*) and optimize *P. aegyptiaca* control efficacy in cabbage fields naturally infested with *P. aegyptiaca*.

### 3.3. Cabbage Response to Herbicide under Field Conditions

#### 3.3.1. Fitted Herbicide Dose for Cabbage Safety

**Figure 3:**
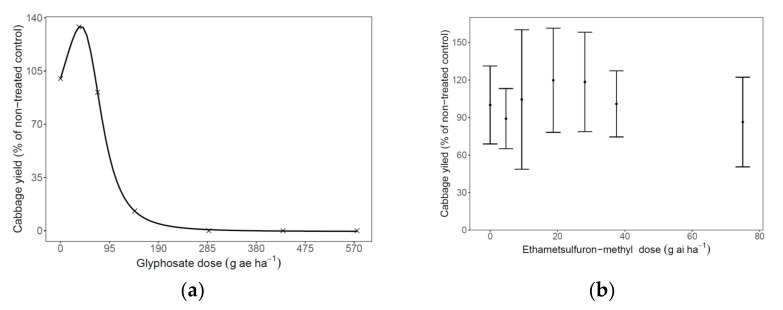
Sensitivity of cabbages to the herbicides glyphosate (**a**) and ethametsulfuron-methyl (EMS, **b**) applied sequentially on 21, 35, and 49 DAP. (**a**) A four-parameter modified sigmoid equation (for u-shaped hormesis) was fitted to the cabbage response to glyphosate; the parameters were as follows: upper asymptote (d = 100, SE = 0.74, *p* ≤ 0.0001), 50% of cabbage yield (X_0_ = 94.83, SE = 0.06, *p* ≤ 0.0001), and size of the hormesis effect (f = 11.63, SE = 0.38, *p* ≤ 0.0001). (**b**) Relationship between cabbage yield and EMS dose. Since there were no significant differences in the response of the cabbages to the various doses of EMS, a regression equation was not fitted.

Experiments A and B were performed in Sde Tzvi to investigate the dose–response effect of glyphosate and EMS on the yield of the host plant. For glyphosate, the sensitivity of the cabbages to the herbicide was reflected by a hormesis effect, which describes the relationship between low doses of glyphosate and cabbage (‘Froctor’) yield (Figure 3a). Three sequential foliar applications to the cabbage at a dose of 72 g ae ha^−1^ glyphosate on 21, 35, and 49 DAP resulted in a slight reduction in cabbage yield (91% compared with the non-treated control). When the hormesis effect was computed at a lower dose of 36 g ae ha^−1^ of glyphosate, the yield increased to 134% compared with the untreated control. For glyphosate doses >72 g ae ha^−1^, the cabbage yield decreased markedly—to as little as 13% compared with the non-treated control. Likewise, cabbage development was inhibited at higher glyphosate doses (Figure 3a). Cabbage yield was not reduced (vs. the non-treated control) for any of the examined sequential applications of EMS (Figure 3b).

#### 3.3.2. Herbicide Applications for Cabbage Save

**Figure 4:**
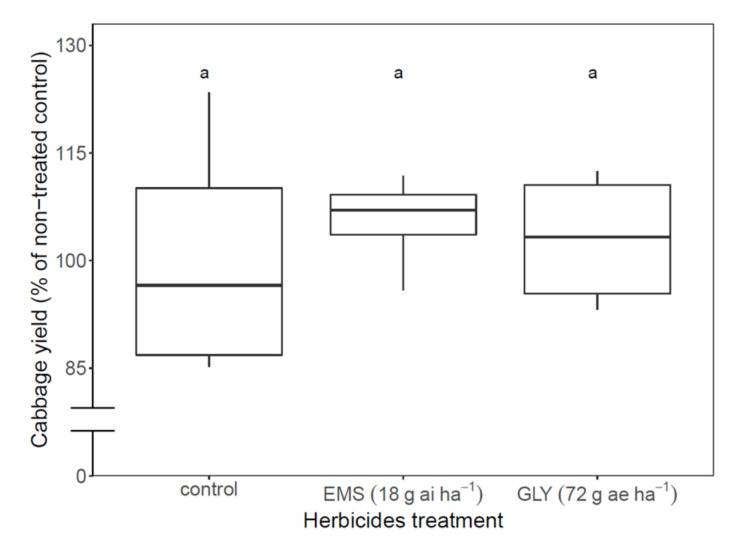
Cabbage sensitivity to the herbicides glyphosate (GLY) and ethametsulfuron-methyl (EMS). Herbicides were applied 21, 35, and 49 DAP at the doses of 72 g ae ha^−1^ glyphosate and 18 g ai ha^−1^ EMS. Means were compared using the Tukey HSD test (*p* < 0.05); means that are indicated with the same letter do not show significant differences.

There was no reduction in cabbage (‘Froctor’) yield in experiment C (Nahalal) when glyphosate or EMS was sequentially applied to the cabbage leaves at the doses of 72 g ae ha^−1^ glyphosate and 18 g ai ha^−1^ EMS; cabbage yields in this field were 103% and 105%, respectively, compared with non-treated control (Figure 4).

### 3.4. Herbicide-Based Control of P. aegyptiaca under Field Conditions

**Figure 5:**
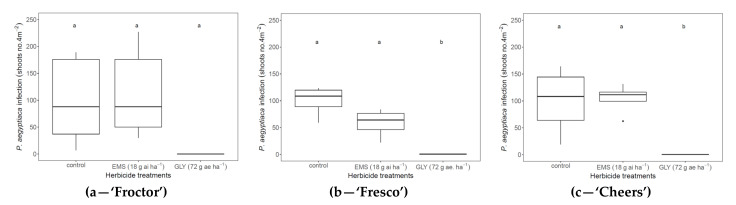
Control efficacy of *P. aegyptiaca* in three cabbage cultivars, ‘Froctor’ (**a**), ‘Fresco’ (**b**), and ‘Cheers’ (**c**). The herbicides glyphosate (GLY) and ethametsulfuron-methyl (EMS) were applied sequentially at doses of 72 g ae ha^−1^ and 18 g ai ha^−1^, respectively, to the cabbage foliage at 21, 35, 49 DAP. Means were compared using the Tukey HSD test (*p* < 0.05); means that are indicated with the same letter do not show significant differences. The experiment was performed in cabbage fields naturally infested with *P. aegyptiaca* at Sde Tzvi (**a**) and Beit Hagedi (**b**,**c**).

To optimize the herbicide dose for *P. aegyptiaca* control in cabbage fields naturally infested with *P. aegyptiaca*, in experiments D, E, and F (Sde Tzvi and Beit Hagedi), glyphosate and EMS were applied at doses of 72 g ae ha^−1^ and 18 g ai ha^−1^, respectively, on the three white cabbage cultivars. When glyphosate was applied to the cabbage foliage, no *P. aegyptiaca* shoots were observed in the field, and full *P. aegyptiaca* control was achieved for all three cultivars (Figure 5a–c). However, EMS was less successful in controlling the parasite, with 114 (‘Froctor’), 58 (‘Fresco’), and 104 (‘Cheers’) *P. aegyptiaca* shoots appearing per 4 m^2^, similar to the non-treated control blocks (Figure 5).

#### Herbicide Adjustment Method Using Overhead Irrigation System

**Figure 6:**
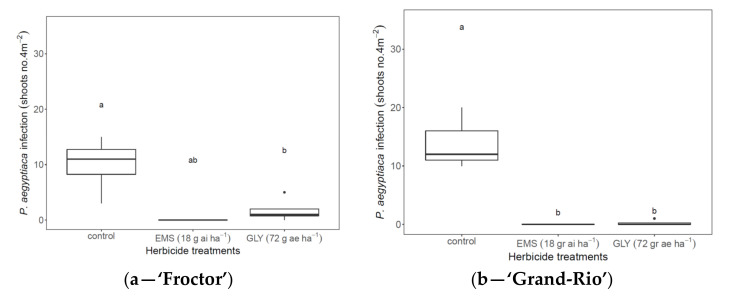
In *P. aegyptiaca*-infested fields, cabbage cultivars ‘Froctor’ (**a**) and ‘Grand-Rio’ (**b**) were treated with three sequential foliar applications of ethametsulfuron-methyl (EMS) or glyphosate (GLY) 21, 35, and 49 DAP at the doses of 72 g ae ha^−1^ glyphosate and 18 g ai ha^−1^ EMS, followed by water spraying after each herbicide eight hours after herbicide application. Means were compared using GLM (*p* < 0.05); means that are indicated with the same letter do not show significant differences.

In experiment G (Mevo Hama), foliar application of the herbicide at doses of 72 g ae ha^−1^ and 18 g ai ha^−1^ for glyphosate and EMS, respectively, was followed by sprinkler irrigation in an amount of 300 m^3^ ha^−1^; the herbicide was thus incorporated into the soil and taken up through the leaves and roots. In fields naturally infested with *P. aegyptiaca*, the herbicides were applied to two cabbage varieties, ‘Froctor’ and ‘Grand-Rio’. No *P. aegyptiaca* shoots appeared when EMS was applied to both varieties. However, when glyphosate was applied, the average numbers of *P. aegyptiaca* shoots counted were 1.754 m^−2^ and 0.254 m^−2^ *P. aegyptiaca* shoots for ‘Froctor’ and ‘Grand-Rio’ cultivars, respectively, compared with 104 m^−2^ and 324 m^−2^ for the non-treated plot, respectively (Figure 6).

## 4. Discussion

The herbicide-based management of *Orobanche* and *Phelipanche* broomrape species in the field is a complicated task because of the unique life cycle and biology of these root parasitic plants. In particular, herbicides that target PSII are not suitable because of the fact that the parasite is lacking a photosynthetic system. Thus, the types of herbicides that can be used are those whose modes of action inhibit the biosynthesis of aromatic (e.g., glyphosate) or branched-chain (e.g., EMS) amino acids [5]. Furthermore, controlling *Phelipanche* in the field must be carried out at the initial soil-subsurface developmental stages, e.g., attachments, tubercle development, and tubercles with crown roots (spider stage) [12,19,23]. Applying herbicides after *Phelipanche* shoots emerge is too late to prevent crop damage, but it is nevertheless effective in preventing seed dispersal because of the sterilizing effect of the herbicide on the broomrape inflorescences [9,11]. There are thus two strategies for herbicide control of *P. aegyptiaca* in the field. The first is to apply the herbicide at the pre-attachment stage directly to the soil so as to prevent attachments or control the small tubercles in the soil sub-surface at the parasitism stage. The second is to apply a systemic herbicide that is translocated via the phloem after the parasite has attached to the host. The latter strategy is based on the functioning of *Phelipanche* species as powerful sinks that draw nutrients and water from the host plant [5].

The EPSPS inhibitor glyphosate is thus usually applied—according to the second strategy—at low doses to the host’s foliage; from there, it moves through the host’s vascular system to the strong broomrape sink [24]. Glyphosate has thus been used for controlling *Phelipanche* infestations in carrot and parsley fields [7,25]. In the current study, when glyphosate was applied to a cabbage field not infested with *P. aegyptiaca*, the cabbage yield was not damaged at doses lower than 72 g ae ha^−1^, with the hormesis effect being detected at 36 g ae ha^−1^. The hormesis response to glyphosate has been shown in other crops, for example, carrot, corn, soy, and barley [7,26,27]. Similar to the reported studies, our results exhibited effective *P. aegyptiaca* control in cabbage when low doses of glyphosate were applied in three sequential applications.

ALS enzyme inhibitors, such as imidazolinones and sulfonylureas, also effectively control species of *Phelipanche*. For example, the imidazolinone herbicides imazapic and imazamox have been used successfully for the control of *P. aegyptiaca* and *O. crenata* in parsley [24], *P. aegyptiaca* in tomato [5], *O. cumana* in sunflower [8,23], and *O*. *minor* in red clover [9]. Among the sulfonylureas, sulfosulfuron is licensed in Israel only for the control of *P. aegyptiaca* in processing tomatoes; it was thus used in a unique DSS for the rational management of the broomrape [11]. Another sulfonylurea, rimsulfuron, has been reported to effectively control *P. aegyptiaca* in potatoes and low infestations in fields of processing tomatoes [6,28]. In this study, yet another member of this group, EMS, was tested for the first time for the control of *P. aegyptiaca* in cabbage. Cabbage sensitivity to EMS was observed at doses higher than the recommended dose (18 g ai ha^−1^).

Under hydroponic conditions (PEB system) in both application methods (foliar application and through the root solution), full control of *P. aegyptiaca* both pre- and post-attachment was observed. In the field, when EMS was applied on cabbage foliage without overhead irrigation (i.e., sprinklers), there was no control of *P. aegyptiaca*. Similar to the current study, Eizenberg et al. reported that sulfonylurea herbicides control *P. aegyptiaca* only when applied to the soil solution [28]. Two reasons may explain the conflicting results obtained with two different application methodologies for EMS. First, in the field, the herbicide was metabolized or excluded, and therefore, it did not reach *P. aegyptiaca*. By contrast, in the PEB system, the cabbage plants remained small, and the herbicide was able to reach the tubercles. Another explanation could be that in the PEB system, the cabbage exudes the herbicide from the roots to the rhizosphere, and the parasite is thus controlled through the soil solution. A similar hypothesis was proposed for the red clover–imazamox association, for which the herbicide was exuded from the roots of red clover to the rhizosphere to control the small broomrape [10].

Applying sulfonylurea herbicides to the soil solution to control the root parasitic plant in the soil sub-surface parasitism stage requires precise knowledge about the parasitism dynamics. Models that predict the parasitism dynamics have been proposed for *P. aegyptiaca* in tomatoes [23], *O. minor* in red clover [29], *O. cumana* in [23], *P. aegyptiaca* in carrot [30], and *O. crenata* in legumes [19]. Thus, the next step in our study of the control of *P. aegyptiaca* in cabbage is the development of the relevant model. In the meantime, the current study has indeed shown that applying EMS via an overhead irrigation system (to incorporate the herbicide into the rhizosphere) prevents the establishment of the parasitic weed in the host root system and guards against yield losses. Moreover, EMS applied at an herbicidal dose also controlled troublesome non-parasitic weeds, whereas a low dose of glyphosate effectively controlled *P. aegyptiaca* alone.

In summary, *P. aegyptiaca* could be controlled using sequential treatments of low glyphosate doses when applied on cabbage foliage and using herbicidal EMS doses when applied on cabbage foliage and incorporated into the soil.

## Data Availability

Not applicable.
